# Analysis of Conformational Determinants Underlying HSP90-Kinase Interaction

**DOI:** 10.1371/journal.pone.0068394

**Published:** 2013-07-02

**Authors:** Rama Krishna Kancha, Natalie Bartosch, Justus Duyster

**Affiliations:** 1 Department Medicine I, University Medical Center Freiburg, Freiburg, Germany; 2 Department of Internal Medicine III, Technical University of Munich, Munich, Germany; University of Geneva, Switzerland

## Abstract

The role of HSP90 in stabilization of oncogenic tyrosine kinases made it an attractive therapeutic target for treating cancer but the molecular basis underlying the interaction between the HSP90 chaperone and client kinases is not elucidated yet. Using kinase inhibitors we show that the inactive conformation of ERBB2 does not interact with HSP90 chaperone and is thus not amenable to degradation upon HSP90 inhibitor treatment, while active ERBB2 kinase conformation promotes interaction with the HSP90 machinery and thus is degraded upon HSP90 inhibitor treatment. Interestingly, the kinase-chaperone interaction is disrupted in case of BCR-ABL and FLT3-ITD when bound to inhibitors irrespective of whether they block the kinase in an active or inactive conformation and thus our results indicate that the stability of the active kinase conformation varies between different kinases.

## Introduction

Targeting HSP90 chaperone has become an important therapeutic possibility to treat cancer due to its importance in oncogenic kinase stabilization [1]. However, the structural basis for HSP90-kinase interaction is not fully elucidated [2]. Interestingly, many mutant oncoproteins are HSP90 clients while their cellular counterparts are not [3]. It has been speculated that a shift from an inactive to an active conformation leads to an association of kinases with the HSP90 chaperone [2], [4]. In fact, it was demonstrated that activating mutations in Src which destabilize the kinase make them dependent on HSP90 for stability [5]. Even though the majority of these HSP90-interacting mutations are activating, mutant kinases with decreased activity when compared to their wild-type counterparts were also reported to be HSP90 clients. For example, B-RAF mutants that have reduced kinase activity displayed enhanced sensitivity towards HSP90 inhibitor mediated degradation [6]. Similarly, kinase-defective ERBB2 remained an HSP90 client indicating that the activation status may not be the sole determining factor for recognition of the client proteins by HSP90 [7].

Previous study indicated a role of surface charge and hydrophobicity as important factors for ERBB2-HSP90 interaction [8]. Thus, the structural details regarding client kinase recognition by HSP90 remained inconclusive. To study the role of kinase conformation as a determinant for client recognition by the HSP90 chaperone, we used a panel of kinase inhibitors that will bind preferentially to either the inactive or active kinase conformation. We show that ERBB2 binds HSP90 only when locked in an active conformation while BCR-ABL and FLT3-ITD disassociate from HSP90 when blocked in an inactive or active conformation by kinase inhibitors.

## Materials and Methods

### Chemical reagents

#### ERBB2 and ALK inhibitors

Erlotinib and lapatinib were purchased from the pharmacy. NVP-TAE-684 and WZ-4002 were purchased from Axon Medchem BV (Groningen, Netherlands). Each compound was dissolved in DMSO to make an initial stock solution of 10 mmol/L (NVP-TAE-684 and WZ-4002) and 2.5 mmol/L (erlotinib and lapatinib).

#### ABL inhibitors

Imatinib mesylate (a kind gift from Novartis pharma AG, Basel, Switzerland) was dissolved in water while nilotinib (a kind gift from Novartis pharma AG, Basel, Switzerland) and dasatinib (a kind gift from Bristol-Myers Squibb Pharmaceutical Research Insitute, Princeton, NJ, USA) were dissolved in DMSO (at 10 mmol/L concentration) and stock solutions were stored at −20°C.

#### FLT3 inhibitors

Sunitinib was purchased from the pharmacy. PKC412 (Midostaurin) was a kind gift from Novartis Pharma AG (Basel, Switzerland). Sorafenib was purchased from American Custom Chemicals Corporation (San Diego, CA, USA). All FLT3 inhibitors were dissolved in DMSO (at 10 mmol/L concentration) and stored at −20°C.

#### HSP90 inhibitors

Geldanamycin and 17-AAG (Tanespimycin) were purchased from InvivoGen, USA. 17-DMAG (Alvespimycin) was purchased from Biozol Diagnostica Vertrieb GmbH, Germany. All HSP90 inhibitors were dissolved in DMSO (at 1 mmol/L for geldanamycin and 17-AAG and at 10 mmol/L for 17-DMAG) and stored at −20°C.

### DNA constructs and cell culture

Ba/F3-ERBB2 [9], Ba/F3-BCR-ABL-WT [10], Ba/F3-BCR-ABL-T315I [10], Ba/F3-FLT3-ITD [11], K562 [12] and KARPAS [13] cells were cultured in RPMI 1640 (Life Technologies) supplemented with 10% FCS and glutamine. FLAG-tagged ERBB2 kinase domain (KD) was cloned into BglII-XhoI sites of MiGR1 vector. Stable Ba/F3 cell line [10] expressing FLAG-tagged kinase domains was generated by retroviral infection and were cultured in the presence of recombinant murine IL-3.

### Immunoprecipitation and western blotting

For immunoprecipitation, Ba/F3 cells expressing wild type ERBB2 were pre-treated with ERBB2 inhibitors for 2 hours followed by treatment with HSP90 inhibitors for 30 minutes. Cells were then lysed in TMNSV buffer [7] (50 mM Tris-HCl pH-7.5, 20 mM Na_2_MoO_4_, 0.09% Nonidet P-40, 150 mM NaCl and 1 mM Sodium orthovanadate) and rabbit anti-ERBB2 antibody (C-18 from Santa-Cruz biotechnology) was used to precipitate ERBB2 protein complexes. K562 cells were treated with ABL inhibitors for 2 hours followed by lysis in TNESV buffer [14] (50 mM Tris-HCl pH-7.5, 2 mM EDTA, 1% NP-40, 20 mM Na_2_MoO_4_, 100 mM NaCl and 10 mM Sodium orthovanadate). Rabbit anti-ABL antibody C-19 (from Santa-Cruz biotechnology) was used to precipitate BCR-ABL protein complexes. KARPAS cells were treated with TAE-684 for 2 hours followed by lysis (TMNSV buffer) and immunoprecipitation with rabbit anti- ALK antibody (C26G7 from Cell Signaling). Ba/F3-FLT3-ITD cells were treated with FLT3 inhibitors for 2 hours followed by immunoprecipitation (TMNSV buffer) with goat anti-FLT3 antibody.

SDS-PAGE and western blotting was performed as described before [9]. Following antibodies were used for immunoblotting: rabbit anti-phpsopho-ERBB2 (Y1221/Y1222 from Santa-Cruz biotechnology), mouse anti-ERBB2 (3B5 from Santa-Cruz biotechnology), mouse anti-ABL (8E9 from BD Biosciences, Heidelberg, Germany), rabbit anti-pFLT3-Y589/Y591 (30D4 from Cell Signaling), mouse anti-pY (4G10 from Upstate Biotechnology and PY10 from BD Biosciences), mouse anti-HSP90 (F-8 from Santa-Cruz biotechnology), mouse anti-Cdc37 (E-4 from Santa-Cruz biotechnology), rabbit anti-NPM1 (Cell Signaling), and mouse anti-CHIP/STUB1 (ST21.55 from Sigma-aldrich). Bands were visualized using the ECL (enhanced chemoluminescence) system (Amersham, Braunschweig, Germany).

### Protein degradation and ubiquitination assays

Ba/F3-ERBB2 cells were pre-treated with ERBB2 inhibitors for 2 hours followed by HSP90 inhibitor treatment for 2 hours. K562, Ba/F3-BCR-ABL-WT and Ba/F3-BCR-ABL-T315I cells were pre-treated with ABL inhibitors for 2 hours before HSP90 inhibitor treatment for 8 hours. KARPAS cells were treated with TAE-684 for 2 hours followed by the treatment with HSP90 inhibitors for 24 hours. Cells were then lysed in cell lysis buffer (10 mM Tris-HCl pH-7.5, 130 mM NaCl, 5 mM EDTA, 0.5% Triton X-100, 20 mM Na_2_HPO_4_/NaH_2_PO_4_ pH-7.5, 10 mM sodiumpyrophosphate pH-7.0, 1 mM Sodiumorthovanadate, 20 mM Sodium fluoride and 1 mM Glycerol-2-phosphate) and subjected to SDS-PAGE and western blotting using indicated antibodies.

For ubiquitination analysis, Ba/F3-ERBB2-WT cells were treated with ERBB2 inhibitors for 2 hours followed by HSP90 inhibitor treatment for 1 hour. Cells were then lysed in cell lysis buffer and subjected to SDS-PAGE and western blotting using rabbit anti-Ub (FL-76 from from Santa-Cruz biotechnology) antibody.

## Results and Discussion

**Figure 1:**
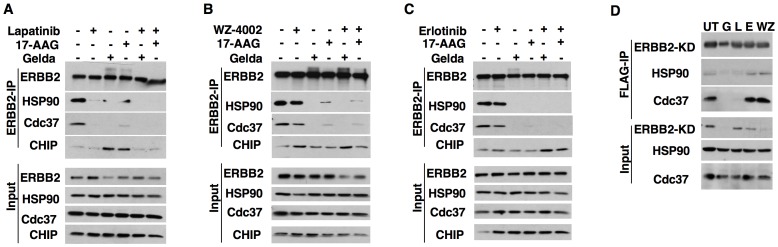
ERBB2 kinase conformation determines its association with HSP90 complex. (A–C) Ba/F3-ERBB2-WT cells[9] were pre-treated with either 1 µM of lapatinib (A), WZ-4002 (B), or erlotinib (C) for 2 hours before HSP90 inhibitor treatment (1 µM of geldanamycin or 17-AAG) for 30 minutes. Immunoprecipitation and immunoblotting was performed with indicated antibodies. (D) Ba/F3 cells[10] stably expressing FLAG-ERBB2-KD were treated with geldanamycin (G), lapatinib (L), erlotinib (E) or WZ-4002 (WZ) for 2 hours followed by cell lysis and immunoprecipitation with anti-FLAG beads.

Wild type ERBB2 is a HSP90 client kinase and was previously shown to be degraded when cells were treated with HSP90 inhibitors [7]. Lapatinib is a type II inhibitor that binds to the ERBB2 kinase domain in an inactive conformation [9], [15]. On the contrary, erlotinib and WZ-4002 are dual EGFR/ERBB2 inhibitors that bind the kinase in an active conformation [15], [16]. Eventhough erlotinib and WZ-4002 are selective EGFR kinase inhibitors, their activity against ERBB2 kinase in sub-micro molar concentrations is well documented [17]–[20]. We first confirmed the inhibitory activity of erlotinb and WZ-4002 towards ERRB2 (Figure S1). We then tested if the kinase-chaperone interaction is intact when ERBB2 is locked in active/inactive conformation by these inhibitors. As reported earlier, HSP90 inhibitor treatment resulted in the disruption of ERBB2-HSP90 and ERBB2-Cdc37 interactions (Figure 1A). Interestingly, lapatinib treatment led to the disruption of both the ERBB2-HSP90 as well as the ERBB2-Cdc37 interactions (Figure 1A). These results indicate that the ERBB2 kinase when in an inactive conformation does not interact with the HSP90. In contrast, treatment with inhibitors that lock the kinase in an active conformation (erlotinib and WZ-4002) didn't disrupt the HSP90-ERBB2 interaction (Figure 1B and Figure 1C). Therefore, these results indicate that the kinase conformation rather than the kinase activity determines their interaction with the chaperone (Figure 1A–C). Furthermore, we observed that the ERBB2-HSP90 interaction remained intact even after the treatment with erlotinib or WZ-4002 at concentrations as high as 5 µM (Figure S1).

To test if kinase domain conformation is the sole determinant of HSP90 interaction, we cloned an FLAG-tagged minimal ERBB2 kinase domain (ERBB2-KD) and tested for its interaction with the HSP90. As observed with the full-length ERBB2 kinase, the ERBB2-KD interacted with HSP90 and this interaction was sensitive to geladanamycin treatment (Figure 1D). Similarly, lapatinib disrupted the interaction of ERBB2-KD with both HSP90 and Cdc37 indicating that these interactions were mediated by the kinase domain (Figure 1D). As expected, erlotinib or WZ-4002 had no effect on the kinase-chaperone interaction (Figure 1D).

**Figure 2:**
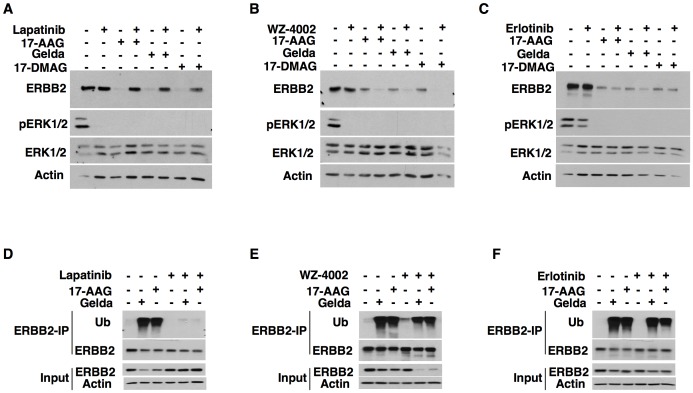
Blocking ERBB2 in an inactive conformation prevents HSP90 inhibitor induced ERBB2 degradation. (A–C) Ba/F3 cells stably expressing wild type ERBB2 (Ba/F3-ERBB2-WT)[9] were pre-treated with either 1 µM lapatinib (A), 1 µM WZ-4002 (B), or 1 µM erlotinib (C) for 2 hours before HSP90 inhibitor treatment (1 µM of geldanamycin, 17-AAG or 17-DMAG) for 2 hours. Cells were then lysed and western blotting was performed to analyze ERBB2 degradation. (D–F) To assess ERBB2 ubiquitination, Ba/F3-ERBB2-WT [9]cells were first treated with 1 µM of lapatinib (D), WZ-4002 (E), or erlotinib (F) for 2 hours followed by HSP90 inhibitor treatment (1 µM of geldanamycin, 17-AAG or 17-DMAG) for 1 hour. Cells were lysed and subjected to ERBB2 immunoprecipitation followed by immunoblotting with indicated antibodies.

To test the effect of inactive kinase conformation on HSP90 inhibitor mediated degradation, cells expressing wild type ERBB2 were incubated with lapatinib prior to treatment with the HSP90 inhibitors geldanamycin, 17-AAG or 17-DMAG. As reported earlier, treatment of ERBB2 expressing cells with HSP90 inhibitors led to the degradation of ERBB2 protein (Figure 2A). However, pre-treatment of these cells with lapatinib which locks the kinase in an inactive conformation abrogated ERBB2 degradation by the HSP90 inhibitors (Figure 2A). In contrast, this effect was not observed with inhibitors that bind ERBB2 kinase in an active conformation (erlotinib and WZ-4002) (Figure 2B and Figure 2C). These results indicate that the inactive kinase conformation prevents while the active kinase conformation allows ERBB2 kinase degradation by different HSP90 inhibitors (Figure 2A–C). Analysis of ERBB2 ubiquitination showed that lapatinib but not erlotinib/WZ-4002 pre-treatment abrogated ERBB2 ubiquitination following HSP90 inhibitor treatment (Figure 2D–F). Thus, this indicates that the ERBB2 ubiquitination following HSP90 inhibitor treatment is influenced by the conformation of the kinase.

**Figure 3:**
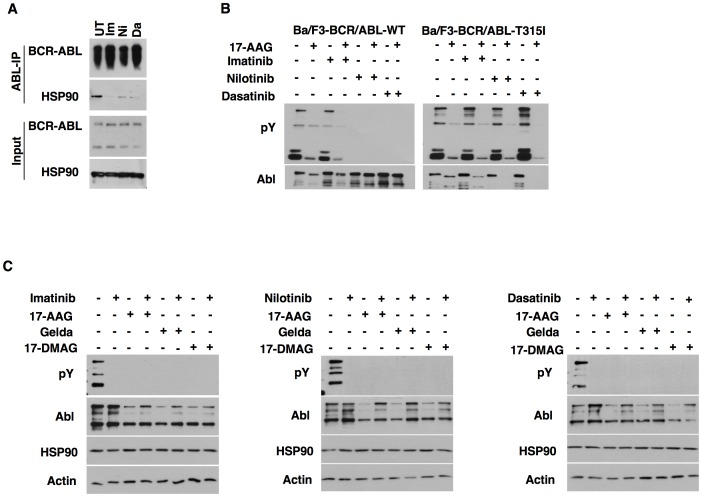
BCR-ABL locked either in an inactive or an active conformation is not degraded by HSP90 inhibitors. (A) K562 cells[12] were treated with 1 µM of imatinib, nilotinib or dasatinib. Cells were then lysed in TMNSV buffer followed by immunoprecipitation and immnoblotting with indicated antibodies. (B) Ba/F3-BCR-ABL-WT[10] (left) or Ba/F3-BCR-ABL-T315I[10] (right) cells were pre-treated with 1 µM of imatinib, nilotinib or dasatinib for 2 hours before 17-AAG (1 µM) treatment for 8 hours. Cells were lysed and subjected to SDS-PAGE and immunoblotting with indicated antibodies. (C) K562 cells[12] were treated with 1 µM of imatinib, nilotinib or dasatinib for 2 hours followed by HSP90 inhibitor treatment (1 µM of geldanamycin, 17-AAG or 17-DMAG) for 8 hours. Cells were lysed and analyzed by immunoblotting with indicated antibodies.

**Figure 4:**
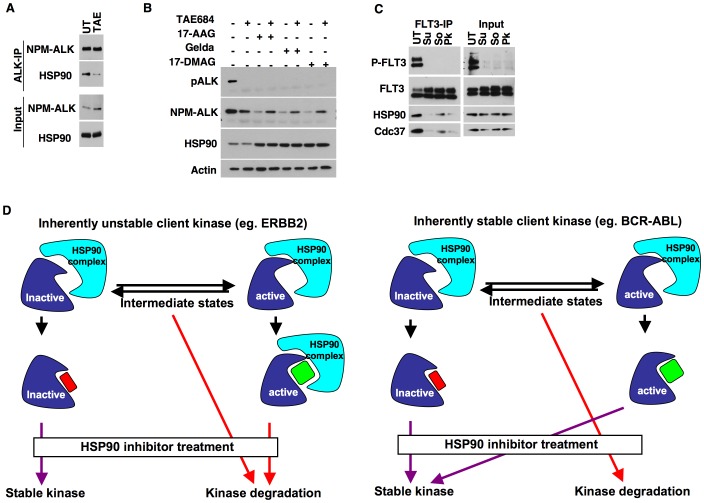
The impact of an active kinase conformation on HSP90 interaction varies between oncogenic tyrosine kinases. (A) KARPAS cells were treated with 0.1 µM of TAE-684 for 2 hours. Cells were then lysed in TMNSV buffer followed by immunoprecipitation and immnoblotting with indicated antibodies. (B) KARPAS cells[13] were treated with 0.1 µM of TAE684 followed by HSP90 inhibitor treatment (1 µM of geldanamycin, 17-AAG or 17-DMAG) for 22 hours. Cells were then lysed and western blotting was performed to analyze NPM-ALK degradation. (C) Ba/F3-FLT3-ITD cells[11] were treated with 1 µM of sunitinib (Su), sorafenib (so) or PKC412 (Pk) for 2 hours. FLT3 immunoprecipitates were then analyzed for the interaction with HSP90 and Cdc37. (D) Possible mechanism underlying client kinase degradation by HSP90 inhibitors.

We further tested if conformation of other oncogenic client kinases effects HSP90 interaction. While imatinib and nilotinib binds BCR-ABL in an inactive kinase conformation, dasatinib binds to the active conformation [21]. Disruption of kinase-HSP90 interaction was observed with BCR-ABL (Figure 3A) when treated with the respective kinase inhibitors that bind either to the inactive (imatinib and nilotinib) or active conformation (dasatinib). Thus, both inactive and active kinase conformations of BCR-ABL are stable in the absence of HSP90 binding. To test the role of kinase conformation in inhibitor mediated degradation, we treated cells expressing BCR-ABL with kinase inhibitors. Interestingly, degradation of BCR-ABL upon HSP90 inhibitor treatment was blocked by pre-treatment of cells with all the ABL kinase inhibitors tested (Figure 3B, left). T315I is a gate-keeper mutation that abrogates inhibitor binding to the BCR-ABL kinase domain. Kinase inhibitors failed to impede BCR-ABL-T315I degradation upon HSP90 inhibitor treatment thus excluding non-specific effects of ABL inhibitors (Figure 3B, right). Similar observations were extended to K562 cells that express BCR-ABL oncoprotein (Figure 3C). We further observed that kinase-HSP90 interactions were disrupted in case of NPM-ALK (Figure 4A) and FLT3-ITD (Figure 4C) irrespective of the mode of inhibitor binding to the kinase. Moreover, pretreatment of KARPAS cells with ALK inhibitor TAE684 rescued the degradation of NPM-ALK upon HSP90 inhibition (Figure 4B).

A recent report showed that when BCR-ABL is blocked either in an inactive or an active conformation by kinase inhibitors, it does not bind HSP90 [22]. In the present study we report similar findings for BCR-ABL and FLT3-ITD. However, interestingly we found that in case of the ERBB2 kinase, only the inactive conformation can displace HSP90 while the ERBB2 kinase in an active kinase conformation still requires HSP90 for its stability. This is evident from the fact that the treatment with HSP90 inhibitors resulted in the degradation of the ERBB2 kinase that is in an inhibitor-induced active conformation. Interestingly, while ERBB2 has a rather short half-life [23], BCR-ABL, NPM-ALK and FLT3-ITD are quite stable proteins [24]–[26]. This observation correlates with differences in the rate of HSP90 inhibitor-mediated degradation: while ERBB2 is completely degraded after 2 hours [8], HSP90 inhibitor-mediated degradation of BCR-ABL, NPM-ALK and FLT3-ITD requires longer times (>8 hours) [14], [27], [28]. These results suggest that the active-conformation of certain kinases (BCR-ABL, NPM-ALK and FLT3-ITD) is more stable which is evident from their slower rate of degradation after HSP90 inhibitor treatment. The stability of certain kinases hereby seems to be independent of chaperone binding since the HSP90-kinase interaction is disrupted very rapidly in all kinases after inhibitor treatment. Taken together, our results suggest that the stability of active conformation varies between different kinases, which may determine their dependence on HSP90 (Figure 4D). This offers a possible explanation for the observed differences in degradation half-lives between different kinases upon HSP90 inhibitor treatment. A recent study by Taipale et al. concluded that the stabilization of the kinase fold and stability determines the client recognition by HSP90. Further analyses of both the inactive and active conformation is required to explain the observed differences in the degradation half-life of different kinases. Moreover, the synergistic effect of the combination of HSP90 and type I kinase inhibitors targeting ERBB2 may be useful potential therapeutic approach for cancer.

## Supporting Information

Figure S1 Effect of erlotinib and WZ-4002 on ERBB2-HSP90 interaction. Ba/F3-ERBB2-WT cells were either untreated (UT) or treated with increasing concentrations (1.0 µM, 2.5 µM or 5.0 µM) of erlotinib (left panel) or WZ-4002 (right panel) for 2 hours. Immunoprecipitation and immunoblotting was performed with indicated antibodies.
(TIF)
