# Supplementary figures and images for: Analysis of Conformational Determinants Underlying HSP90-Kinase Interaction
Source: PLoS One. 2013 Jul 2;8(7):e68394. doi: 10.1371/journal.pone.0068394 (PMC3699556; doi:10.1371/journal.pone.0068394)

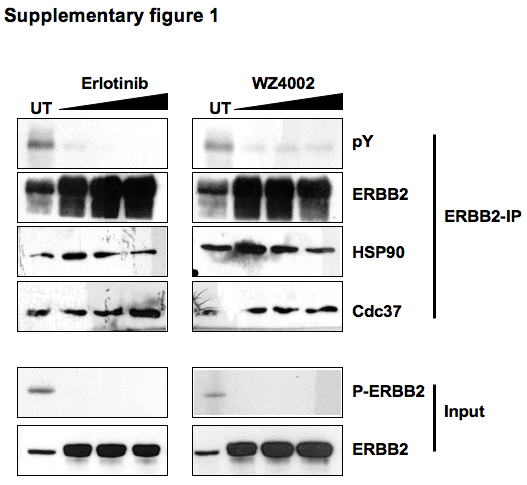

Supplement: Figure S1 — Effect of erlotinib and WZ-4002 on ERBB2-HSP90 interaction. Ba/F3-ERBB2-WT cells were either untreated (UT) or treated with increasing concentrations (1.0 µM, 2.5 µM or 5.0 µM) of erlotinib (left panel) or WZ-4002 (right panel) for 2 hours. Immunoprecipitation and immunoblotting was performed with indicated antibodies. (TIF) [file pone.0068394.s001.tif]
